# Personality and Nomophobia: The Role of Dysfunctional Obsessive Beliefs

**DOI:** 10.3390/ijerph20054128

**Published:** 2023-02-25

**Authors:** Victoria García-Masip, Beatriz Sora, Maria José Serrano-Fernandez, Joan Boada-Grau, Bettina Lampert

**Affiliations:** 1 Department of Psychology, Faculty of Education Sciences and Psychology, University Rovira i Virgili, Carretera de Valls, s/n, 43007 Tarragona, Spain; 2 Department of Psychology, University of Innsbruck, 6020 Innsbruck, Austria

**Keywords:** nomophobia, dysfunctional obsessive beliefs, personality traits

## Abstract

Background: The development of new technologies (ICTs), and specifically the invention of smartphones, has offered users enormous benefits. However, the use of this technology is sometimes problematic and can negatively affect people’s lives. Nomophobia has been defined as the fear of being unreachable by means of a smartphone and is considered a disorder of the modern world. The present study aims to provide additional evidence of the relationship between personality traits and nomophobia. Moreover, this research explores dysfunctional obsessive beliefs as another possible antecedent. Finally, this study also examines the effect of the combination of these antecedents on nomophobia. Method: The study sample was comprised of Spanish workers (males: 44.54%; females: 55.46%) in the city of Tarragona and its surroundings. Results: Our results showed that nomophobia is directly related to personality traits such as extraversion, and that dysfunctional obsessive beliefs play a role in the development of nomophobia. Moreover, our study confirms that the combination of personality traits and dysfunctional obsessive beliefs can affect the degree of nomophobia experienced. Discussion and Conclusions: Our study contributes to the body of literature that examines how psychological variables of personality can be predictors of nomophobia. Additional research is needed to better understand the determinants of nomophobia.

## 1. Introduction

### 1.1. Personality and Nomophobia: The Role of Dysfunctional Obsessive Beliefs

Lifestyles have changed drastically in recent years, among other reasons, because of the rapid development of information and communication technologies (ICTs). ICTs, such as smartphones, have become an indispensable part of our lives [1,2] because they involve numerous benefits for users; for example, to perform a variety of daily tasks with a single device, [3], and meet some of their needs. In contrast, smartphones also present some risks for health. The indiscriminate use of smartphones can provoke psychological disorders [4]. In fact, a specific phobia related to smartphones has been included within the phobic group of anxiety disorders: nomophobia. The term is a portmanteau of ‘no mobile phone phobia’ [5,6]; it is defined as the ‘irrational’ fear of not being able to use one’s smartphone, and it is characterized by four dimensions [3]. (1) Fear of not being able to communicate refers to feelings of losing instant communication with people (2) Losing connectedness is related to feelings of losing the ubiquitous connectivity that smartphones provide and being disconnected from one’s online identity (3) Not being able to access information reflects the discomfort of losing pervasive access to information and to search for things on smartphones. Lastly, (4) giving up convenience reflects the desire to take advantage of the convenience of having a smartphone.

Given that nomophobia is a relatively new phenomenon, much remains to be known. Yildirim and Correia (2015) [3] called for further research to clarify the predicting factors of nomophobia. In this regard, some studies have focused on identifying specific antecedents, such as sociodemographic variables (e.g., age or gender) [7,8,9], occupation [8], monthly income [10] and personality traits [11,12].

However, research into the association between personality and nomophobia has yielded non-conclusive, incongruent and variable results. Some studies have shown strong relationships between personality traits and nomophobia (e.g., [7,13]), whereas others have found weak (e.g., [14]), or even nonsignificant relationships (e.g., [7,11,15,16]). In general, research has explained this variability by suggesting that personality-psychological disorders can be influenced by other factors, such as dysfunctional beliefs (e.g., [17,18]).

This study aims to provide additional empirical evidence in an attempt to clarify the relationships between the different dimensions of personality according to the Big Five model (extraversion, agreeableness, emotional stability, conscientiousness and openness to experience), obsessive beliefs and nomophobia.

### 1.2. Personality: The Big Five Model

Personality reflects the ways of thinking, feeling and behaving that define an individual [19,20]. One of the main theoretical models used to represent personality is the Big Five model [21,22,23], which describes personality as a hierarchical model with five general traits [24,25]. (1) Extraversion (vs. introversion) refers to the traits of being sociable, gregarious, assertive, talkative, and active. (2) Neuroticism (vs. emotional stability) is characterized by being anxious, depressed, emotional, worried, insecure, frustrated, irritable and by having difficulty controlling impulses and desires. (3) Agreeableness (vs. hostile non-compliance) refers to being courteous, flexible, trusting, cooperative, relaxed, having emotional stability, and being open to criticism. (4) Conscientiousness (vs. lack of direction) is defined as “a spectrum of constructs that describe individual differences in the propensity to be self-controlled, responsible to others, hardworking, orderly, and rule abiding” [26] (p. 1). Finally, (5) openness to experience (vs. closedness) includes the traits of being broad-minded, intelligent, and artistically sensitive [27].

The link between personality and psychological disorders has been extensively studied and demonstrated over the years (see, for example, the meta-analysis by Kotov et al., 2010) [28]. Regarding nomophobia, this connection is not so clear. Some studies have found positive and significant relationships between extraversion [7,13] and/or neuroticism [7,13,29] and nomophobia. These studies suggest that technologies may affect people’s behaviors, mood and emotions. For example, smartphones promote enjoyable feelings [30]. Hence, extraversion, as a trait related to excitement, stimulation, action and thrills, was positively associated with nomophobia. For extrovert people, anything that alters this state of enjoyment could cause anxiety. Similarly, neuroticism pertains to fluctuations in emotions and is associated with dispositions as anxiety, impulsiveness and self-consciousness [31]. Neurotic individuals tend to demonstrate sensitivity and vulnerability to their social environment [20]. Hence, a lack of sources of enjoyment, such as a smartphone, may affect them more than other people with higher emotional stability.

Other studies have also found a significant but negative relationship between conscientiousness [7], agreeableness [15] nd/or openness to experience and nomophobia [13,15]. These studies suggest that conscientious people may develop and employ strong self-control mechanisms that determine their smartphone use, whereas unconscientious individuals tend to be disorganized people who act impulsively. Hence, conscientiousness was determined to have a negative relationship with nomophobia. In addition, disagreeable individuals are more likely to develop personality disorders, such as antisocial disorders [32]; therefore, agreeableness also presented a negative association with nomophobia. Finally, smartphones allow people to easily convey their feelings, ideas and thoughts. This is especially useful for individuals with low openness to experience, who have greater difficulty expressing themselves. The relationship between openness to experience and nomophobia was also found to be negative.

In contrast, some studies have also found nonsignificant relationships between personality traits and nomophobia. For example, Prasetyo et al. (2016) [16] reported a nonsignificant relationship between the Big Five personality traits and nomophobia. Similarly, Buckner, Castille and Sheets (2012) [11] found nonsignificant relationships between extraversion, neuroticism and nomophobia. Argumosa-Villar et al. (2017) [7] reported nonsignificant associations between agreeableness, openness to experience and nomophobia. Finally, Andreassen et al. (2013) [15] and Yoğurtçu (2018) [13] showed that the association between agreeableness, conscientiousness and nomophobia were also nonsignificant.

Given these incongruent results, this research aims to provide additional empirical evidence to clarify the relationship between personality traits and nomophobia. We therefore posed the following hypotheses:

**Hypothesis** **1.** *Extraversion is positively related to nomophobia.*

**Hypothesis** **2.** *Emotional stability is negatively related to nomophobia.*

**Hypothesis** **3.** *Conscientiousness is negatively related to nomophobia.*

**Hypothesis** **4.** *Agreeableness is negatively related to nomophobia.*

**Hypothesis** **5.** *Openness to experience is negatively related to nomophobia.*

### 1.3. Obsessive Beliefs

Dysfunctional beliefs are based on the cognitive theory [33], which suggests that people suffer due to their interpretation of events more than the events themselves. So, dysfunctional beliefs reflect an association between specific cues and catastrophic consequences or states [34]. In fact, psychopathology may underpin certain cognitive variables, such as the type of beliefs and/or specific interpretations that each person holds about their intrusive thoughts [35].

A relevant amount of literature has supported this assumption, especially for phobias. For example, Gellatly (2016) [36] showed how catastrophic thinking may play a critical role in a variety of disorders by being a predictor of psychopathological disorders, including phobias. Thorpe et al. (1995) [37] suggested that idiosyncratic cognitions may be primary to the experience of phobic anxiety. Harm cognitions were strongly related to some phobias. Ollendick et al. (2017) [38] stated that catastrophic beliefs and low coping expectancies are often present in people with specific phobias. Stopa and Clark (1993) [39] concluded that socially phobic individuals are characterized by specific dysfunctional beliefs (negative self-evaluative thoughts).

Another possible dysfunctional belief associated with phobias may be obsessive beliefs. Several authors (e.g., [40,41]) have proposed that dysfunctional obsessive beliefs are not exclusive to obsessive-compulsive disorder but may also be determining factors in other psychological disorders. Dysfunctional obsessive beliefs reflect intrusive, recurrent thoughts that come spontaneously to the mind and are unpleasant (scary, distressing or disturbing). Based on the study by Belloch et al. (2010) [40], we focused on these two dysfunctional obsessive beliefs: perfectionism, that refers to the belief that there is a perfect solution for every problem, and that making it perfect is possible and necessary, so that any failure will have serious consequences; and excessive responsibility, that reflects the belief that one can cause, and therefore should prevent, major negative events, which leads the person to feel very overly responsible for everything that happens.

Despite the growing research on dysfunctional obsessive beliefs and phobias, we are not aware of any study that has specifically examined the relationship between dysfunctional obsessive beliefs and nomophobia. To our knowledge, only two studies have linked obsessiveness to nomophobia. Lee et al. (2018) [42] found that high levels of obsessiveness were associated with high levels of nomophobia. Adawi et al. (2019) [43] reported that obsession-compulsion was positively related to nomophobia, understood in terms of not being able to access information, giving up convenience/losing connectedness, and not being able to communicate. According to the research on dysfunctional beliefs and phobias, it seems plausible to suggest that dysfunctional obsessive beliefs may play a role in degrees of nomophobia. We therefore hypothesized the following.

**Hypothesis** **6.** *Dysfunctional obsessive beliefs are positively related to nomophobia.*

### 1.4. The Combination of Personality and Obsessive Beliefs

A considerable body of research has shown that the combination of personality traits and dysfunctional beliefs may result in detrimental outcomes to people’s mental health. For example, McDermut et al. (2019) [17] found that dysfunctional obsessive beliefs play a critical role in emotional distress in individuals with personality dysfunction. Dysfunctional obsessive beliefs mediated the relationship between the Big Five personality dimensions and the psychopathology dimensions (positive emotionality, psychoticism, aggressiveness, and disconstraint). The mediation analyses indicated that personality variables operated through dysfunctional obsessive beliefs to exert their effect on other outcomes. Similarly, Zahura (2020) [18] also demonstrated that personality dysfunction, dysfunctional obsessive beliefs, and negative emotional outcomes are closely related. Unlike McDermut et al. (2019) [17], Zahura (2020) [18] found that negative emotional outcomes were predicted by the interaction of personality and dysfunctional obsessive beliefs, and not by a mediation effect. In fact, Zahura (2020, p. 4) [18] explained that the “moderation for the dimensions of negative emotions, depression, social anxiety and anger since it’s more appropriate to support that connection, because with mediation too many assumptions are made that can’t be easily justified”. So, these authors concluded that moderation makes more sense to explain these relationships because it can identify subgroups who are the most at risk of negative emotional outcomes (depression, anxiety, anger, etc.).

Taking into account the relevant literature on the relationship between personality and nomophobia, the research on dysfunctional obsessive beliefs and phobias, and the study by Zahura (2020) [18], which reported that the combination of personality and dysfunctional obsessive beliefs can explain cognitive and emotional outcomes, it seems plausible to propose that personality and dysfunctional obsessive beliefs may interact to explain nomophobia, despite the apparent lack of studies examining the possible combination of personality traits and dysfunctional obsessive beliefs to explain the disorder. Hence, the present work aimed to examine the moderating role of obsessive beliefs in the relationship between personality traits and nomophobia. We therefore posed the following hypotheses.

**Hypothesis** **7.** *Obsessive beliefs moderate the relationship between extraversion and nomophobia.*

**Hypothesis** **8.** *Obsessive beliefs moderate the relationship between emotional stability and nomophobia.*

**Hypothesis** **9.** *Obsessive beliefs moderate the relationship between conscientiousness and nomophobia*.

**Hypothesis** **10.** *Obsessive beliefs moderate the relationship between agreeableness and nomophobia.*

**Hypothesis** **11.** *Obsessive beliefs moderate the relationship between openness to experience and nomophobia.*

## 2. Methods

### 2.1. Procedure

Non-probabilistic sampling [44] was used to obtain the samples. Assistant researchers collected data through their personal contacts. They described the research project and asked for participation. Those who volunteered to participate completed the questionnaire. The volunteers were assured of data confidentiality and anonymity. Accordingly, a wholly random sampling method was not possible, given the reliance on voluntary participation.

The study was conducted in accordance with the Declaration of Helsinki, and the protocol followed the guidelines of the Ethics Committee of our university.

### 2.2. Participants

The sample was made up of 366 working people from the city of Tarragona (Spain), 44.54% of whom were men (*n* = 163) and 55.46% of whom were women (*n* = 203). Participant age varied from 18 to 99 years old (mean = 40.43; SD = 13.318); 55.5% of the sample were married (*n* = 203), 35% single (*n* = 128), 8.5% divorced (*n* = 31) or separated and 1.1% were widowed (*n* = 4); 1.4% of the total sample had no formal education (*n* = 5), 7.9% had completed primary school (*n* = 29), 57.9% had completed secondary school (*n* = 212), 22.1% had university studies (*n* = 81) and 10.7% had post-graduate studies (*n* = 39).

### 2.3. Measures

Nomophobia was measured using the Nomophobia Questionnaire (NMP-Q) [3], which consists of twenty seven point Likert items ranging from 1 (strongly disagree) to 7 (strongly agree). The scale contained four dimensions: (1) Not being able to communicate (six items; e.g., “I would feel nervous because I would not be able to receive text messages and calls”; α = 0.94); (2) Losing connectedness (five items; e.g., “I would be uncomfortable because I could not stay up-to-date with social media and online networks”, α = 0.94); (3) Not being able to access information (four items; e.g., “I would be annoyed if I could not use my smartphone and/or its capabilities when I wanted to do so”, α = 0.94); (4) Giving up convenience (four items; e.g., “Running out of battery in my smartphone would scare me”, α = 0.94).

Personality traits were measured using the Overall Personality Assessment Scale (OPERAS) [45], an instrument based on the Big Five model of personality factors. The scale contains a total of 40 items, with a five-point Likert scale ranging from 1 = strongly disagree to 5 = strongly agree. The Cronbach’s alphas were the following: extraversion (EX) (0.86; e.g., “I make friends easily”), emotional stability (ES) (α = 0.86; e.g., “I often feel sad”), conscientiousness (CO) (α = 0.77; e.g., “I am a perfectionist”), agreeableness (AG) (α = 0.71; e.g., “I am often unpleasant with others”) and openness to experience (OE) (α = 0.81; e.g., “I like to visit museums”).

Dysfunctional obsessive beliefs were assessed using a global measure of its two main dimensions: perfectionism and intolerance of uncertainty and excessive responsibility and importance of controlling thoughts. It was measured through the Inventory of Obsessive Beliefs (ICO) [35], with a scale of 24 items, with response options that ranged from 1 (strongly disagree) to 7 (strongly agree). The perfectionism and intolerance of uncertainty dimension consisted of 14 items. An example item was “I must be the best in what is important to me”. The excessive responsibility and importance of controlling thoughts dimension consisted of 10 items; for example, “I should be able to rid my mind of inappropriate thoughts”. The Cronbach’s alpha was 0.92.

### 2.4. Analysis

After the preliminary analyses (descriptive analysis and correlations) were conducted, three hierarchical multiple regression analyses were performed to test our hypotheses. In line with that reported in Cohen and Cohen (1983) [46], the lower-order variables were introduced first and the higher-order terms later. The control variables (sex and age) were entered in step 1. In step 2, the predictor variables (personality traits and dysfunctional obsessive beliefs) were introduced, and in step 3, interaction terms among variables were entered. We used centered scores to solve the possible problem of multicollinearity and to maximize interpretability. Finally, a graphic representation was generated to better understand the nature of the interactions [47].

## 3. Results

Table 1 presents the means, standard deviations and correlations among the variables. Most of variables were significantly related. The correlation values ranged from −0.12 to 0.51.

The results of the regressions are presented in Table 2. They show a positive and significant relationship between extraversion and nomophobia. Hypothesis 1 was therefore supported. Hypothesis 2 was partially supported. Our results show negative and significant relationships between emotional stability and the fear of not being able to communicate by means of a smartphone, and not being able to access information. However, the relationships between emotional stability and losing connectedness and giving up convenience were nonsignificant. Hypothesis 3 was also partially supported. Conscientiousness was only negatively related to fear of losing connectedness. Hypothesis 4 was rejected. Nonsignificant relationships were found between agreeableness and nomophobia. Finally, hypothesis 5 was supported. Openness to experience was negatively related to the fear of not being able to communicate by means of a smartphone, losing connectedness, and not being able to access information and giving up convenience.

The relationship between dysfunctional obsessive beliefs and nomophobia was supported as stated in hypothesis 6. Our results show a positive and significant relationship between dysfunctional obsessive beliefs and the four dimensions of nomophobia.

**Figure 1:**
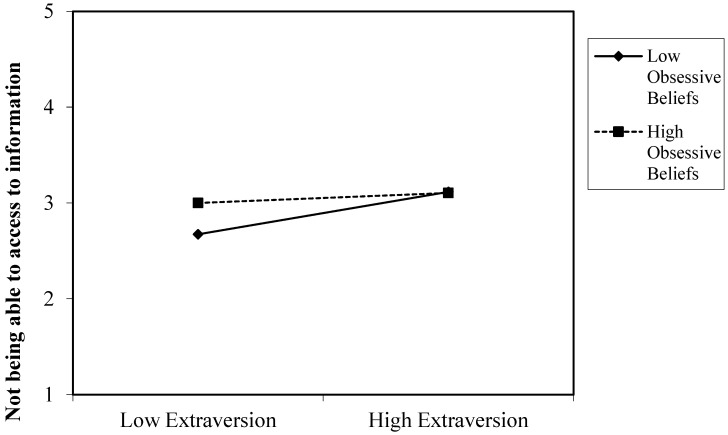
Interaction of extraversion and obsessive beliefs in predicting fear of not being able to access information.

Table 2 also shows the significant interaction effect on nomophobia. Our results partially supported hypothesis 7, as we found a significant interaction between extraversion and obsessive beliefs to predict the fear of not being able to access information. Figure 1 is the plot of interaction between extraversion and dysfunctional obsessive beliefs in predicting the fear of not being able to access information. When obsessive beliefs are high, fear of not being able to access to information do not seem to vary, regardless of the level of extraversion. Extroverted and introverted people reported similar levels of fear of not being able to access information when they presented high levels of obsessive beliefs. However, among subjects with a low degree of obsessive beliefs, differences between extroverts and introverts were detected in the level of fear of not being able to access information. People with higher extraversion are more fearful of not being able to access information by means of a smartphone than those with low extraversion (introverts).

**Figure 2:**
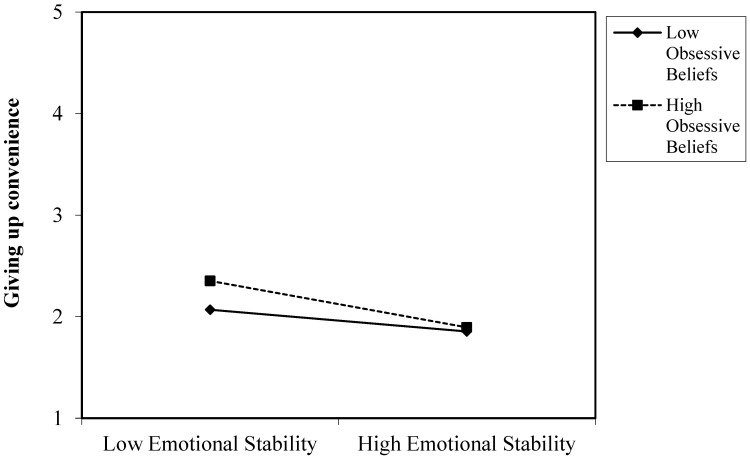
Interaction of emotional stability and obsessive beliefs in predicting the fear of giving up convenience.

Figure 2 presents the graphic representation of the interaction between emotional stability and dysfunctional obsessive beliefs in predicting the fear of giving up convenience, therefore partially supporting hypothesis 8. People with high emotional stability exhibited similar levels of discomfort at giving up convenience regardless of whether or not they had obsessive beliefs. However, when they reported lower levels of emotional stability, they experienced differences in the fear of giving up convenience depending on the degree of their obsessive beliefs. More specifically, people with low emotional stability and high dysfunctional obsessive beliefs showed a greater fear of giving up convenience than people with lower degrees of obsessive beliefs.

**Figure 3:**
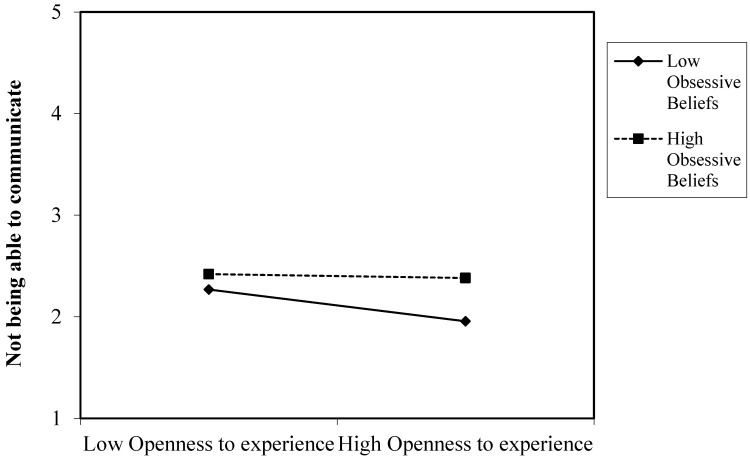
Interaction of openness to experience and obsessive beliefs in predicting the fear of not being able to communicate.

**Figure 4:**
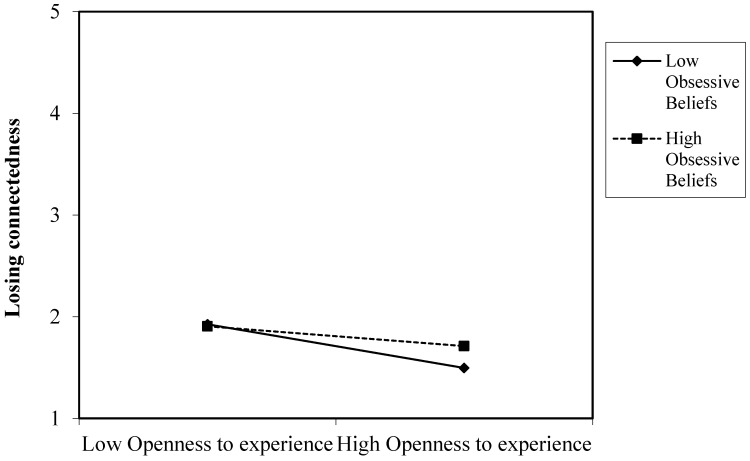
Interaction of openness to experience and obsessive beliefs in predicting the fear of losing connectedness.

**Figure 5:**
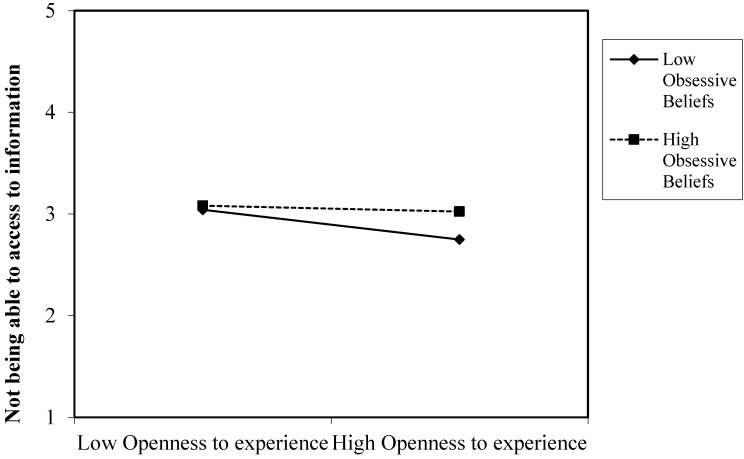
Interaction of openness to experience and obsessive beliefs in predicting the fear of not being able to access information.

Our results partially supported hypothesis 11 by showing that dysfunctional obsessive beliefs moderate the relationships between openness to experience and the fear of not being able to communicate, losing connectedness and not being able to access information. Figure 3, Figure 4 and Figure 5 present the plot of the interaction between openness to experience and dysfunctional obsessive beliefs in predicting the fear of not being able to communicate by means of a smartphone, losing connectedness and not being able to access information, respectively. These three figures present similar results. People with high degrees of obsessive beliefs along with a high openness to experience exhibited a similar degree of fear of not being able to communicate, losing connectedness and access to information to those with low openness to experience. However, people with low degrees of obsessive beliefs along with low openness to experience exhibited higher levels of fear of not being able to communicate, losing connectedness and access to information compared to people with high openness to experience.

## 4. Discussion

The smartphone has become an indispensable resource in people’s lives. However, it has also brought about negative consequences for some people. Among them, a new phobia has emerged in our society: nomophobia. Nomophobia reflects the fear of a lack of access to technology for communication or access to information [48,49,50,51]. This phenomenon is relatively new, and it warrants further research to better understand it. Hence, the present study aimed to examine how personality traits, dysfunctional obsessive beliefs and the combination of the two may predict nomophobia.

The first contribution of this study is that it provides additional evidence regarding the association between personality traits and nomophobia. Our results show that extraversion is positively related to all four dimensions of nomophobia. The more extraverted a person was, the greater his or her fear of not being able to communicate, losing connectedness, not being able to access information and giving up convenience. Emotional stability was negatively related to the fear of not being able to communicate by means of a smartphone and not being able to access information. More emotionally stable people presented lower levels of nomophobia, in terms of the dimensions of fear of not being able to communicate by means of a smartphone and not being able to access information. Conscientiousness was only negatively related to the fear of losing connectedness; thus, more conscientious people exhibit less fear of losing connectedness. Finally, an openness to experience was negatively related to all four dimensions of nomophobia. People who were more open to experiences presented lower degrees of nomophobia than those with a lower openness to experience. Consequently, it seems plausible to conclude that extraversion and openness to experience are the most critical personality traits in the development of nomophobia, whereas agreeableness is not associated with nomophobia. All results are consistent with those of previous studies on the association between personality and nomophobia [33,52,53], but also with those that found nonsignificant associations between agreeableness and nomophobia (e.g., [7,11,15]).

The second contribution of this study is that it sheds light on the potential association between dysfunctional obsessive beliefs and nomophobia. Our results show that dysfunctional obsessive beliefs are positively related to all dimensions of nomophobia. People with dysfunctional obsessive beliefs presented higher levels of nomophobia. This finding is consistent with the cognitive theory [33] which suggests that people with dysfunctional beliefs associate specific events to detrimental consequences, which may underpin psychological disorders. These results are also congruent with the empirical research that has demonstrated the relationship between dysfunctional beliefs and phobias (e.g., [36,37,38,39])., and the growing research that has shown a link between obsessiveness and nomophobia (e.g., [42,43]).

The third contribution of this study is that it shows how obsessive beliefs might explain the variability in the research on the association between personality traits and nomophobia. Dysfunctional obsessive beliefs might sustain levels of nomophobia, regardless of personality traits. Subjects who presented high levels of obsessive beliefs experienced higher levels of nomophobia irrespective of their personality traits. In other words, extroverts and introverts exhibit a similar fear of not being able to access information if they have high dysfunctional obsessive beliefs. Likewise, both people open to experiences and those who are not open to experiences presented similar levels of fear of not being able to communicate, losing connectedness and not being able to access information by means of a smartphone. An exception was the relationship between emotional stability and giving up convenience. People with low emotional stability and high levels of obsessive beliefs experienced a greater fear of giving up convenience compared to people with higher emotional stability. In this vein, low compulsive beliefs moderated the relationship between personality traits and nomophobia. So, people who are extroverted, emotionally unstable, and not very open to experience exhibited higher levels of nomophobia than people who are introverted, emotionally stable and open to experience when they also had low obsessive beliefs. These results are congruent with incipient research suggesting that dysfunctional obsessive beliefs may intervene in the relationship between personality traits and psychological disorders (e.g., [17,18]).

Although these results are very interesting, they should also be interpreted with caution, bearing in mind the potential limitations of this study. First, the sample was collected in a specific region of Spain. Thus, results must be extrapolated to other populations in caution. Further research is warranted to provide additional empirical evidence to these research objectives. Second, causal relationships between variables cannot be inferred because a cross-sectional design was used. The literature on nomophobia includes few longitudinal studies that test causal relationships over time. More research is needed in this area. Another possible limitation is that all our variables were assessed by means of self-reported questionnaires. Hence, the results may be influenced by common-method variance. Other methods could be applied to collect data in future research. Finally, we controlled the most critical external factors that might affect our results (i.e., sex and age), but other unmeasured variables could also influence the relationship between personality, dysfunctional obsessive beliefs and nomophobia. Additional research is warranted.

These results also carry several theoretical and practical implications. Our study contributes to the body of literature that examines how personality traits and dysfunctional obsessive beliefs may be related to nomophobia. Specifically, it highlights the moderating role of obsessive beliefs in the personality-nomophobia link to explain the variability among studies. The practical implications of this study include the proposal that interventions for nomophobia may require personalization and individualization focused on dysfunctional obsessive beliefs.

Finally, our study examined a series of psychological factors that might explain nomophobia. However, additional research is needed to better understand the determinants of nomophobia. For example, social factors could also influence nomophobia (i.e., social and family relationships). Finally, longitudinal studies are warranted to established causal relationships between these determinants and nomophobia.

## 5. Conclusions

This study evidenced how personality traits and dysfunctional obsessive beliefs, and their interaction, are associated to nomophobia, taking into account its different dimensions. Specifically, it highlights the moderating role of obsessive beliefs in the relationship between personality traits and nomophobia. This study contributed to advance the knowledge about nomophobia with important practical implications.

## Figures and Tables

**Table 1:** Descriptive analysis (mean and standard deviation) and correlations.

	Mean	SD	1	2	3	4	5	6	7	8	9	10	11	12
1. Sex	1.55	0.498	-											
2. Age	40.43	13.318	−0.121 *	-										
3. Extraversion	49.336	9.486	−0.001 *	−0.099 *	-									
4. Emotional stability	48.633	11.477	−0.173 **	0.099	0.443 **	-								
5. Conscientiousness	48.300	11.753	0.021	0.145 **	0.291 **	0.517 **	-							
6. Agreeableness	49.418	10.468	0.062	066	0.144 **	0.427 **	0.395 **	-						
7. Openness to experience	50.019	9.513	0.089	0.094	0.214 **	0.302 **	0.301 **	0.351 **	-					
8. Obsessive beliefs	0.000	0.936	0.015	−0.046	0.053	0.169 **	0.376 **	0.035	−0.050	-				
9. Not being able to communicate	2.830	0.999	0.240 **	−0.107 *	0.043	−0.187 **	−0.031	−0.132 *	−0.195 **	0.264 **	-			
10. Losing connectedness	1.937	0.885	0.094	−0.114 *	0.019	−0.171 **	−0.183 **	−0.155 **	−0.283 **	0.065	0.617 **	-		
11. Not being able to access information	3.062	1.022	0.079	−0.226 **	0.130 *	−0.108 *	−0.062	−0.049	−0.159 **	0.134 *	0.537 **	0.495 **	-	
12. Giving up convenience	2.572	0.977	0.222 **	−0.211 **	0.014	−0.214 **	−0.146 **	−0.201 **	−0.275 **	0.140 **	0.710 **	0.625 **	0.632 **	-

* *p* < 0.05; ** *p* < 0.001.

**Table 2:** Hierarchical multiple regression analysis of personality traits and obsessive beliefs in predicting nomophobia.

	Not Being Able to Communicate			Losing Connectedness			Not Being Able to Access Information			Giving up Convenience		
	B	SE	*p*	B	SE	*p*	B	SE	*p*	B	SE	*p*
Step 1												
Sex	0.230	0.103	0.000	0.082	0.093	0.120	0.052	0.106	0.309	0.200	0.099	0.000
Age	−0.079	0.051	0.122	−0.105	0.046	0.047	−0.219	0.053	0.000	−0.187	0.049	0.000
Step 2												
Extraversion	0.158	0.054	0.004	0.142	0.050	0.013	0.216	0.058	0.000	0.123	0.053	0.024
Emotional stability	−0.191	0.064	0.003	−0.082	0.059	0.225	−0.161	0.069	0.017	−0.110	0.063	0.088
Conscientiousness	−0.031	0.063	0.623	−0.146	0.058	0.026	−0.060	0.067	0.359	−0.080	0.061	0.206
Agreeableness	−0.029	0.056	0.601	−0.005	0.052	0.930	0.066	0.060	0.264	−0.075	0.055	0.180
Openness to experience	−0.156	0.053	0.003	−0.243	0.049	0.000	−0.143	0.057	0.010	−0.217	0.052	0.000
Obsessive beliefs (OB)	0.289	0.052	0.000	0.111	0.048	0.043	0.155	0.056	0.005	0.165	0.051	0.002
Step 3												
ExtraversionxB	−0.060	0.060	0.285	0.017	0.056	0.766	−0.152	0.065	0.010	0.006	0.059	0.913
Emotional stabilityxOB	−0.151	0.050	0.110	−0.099	0.047	0.317	−0.081	0.054	0.414	−0.233	0.050	0.014
ConscientiousnessxOB	0.089	0.060	0.288	−0.035	0.055	0.691	0.088	0.064	0.314	0.101	0.059	0.230
AgreeablenessxOB	−0.008	0.058	0.914	0.029	0.054	0.721	0.008	0.062	0.922	0.105	0.057	0.172
Openness to experiencexOB	0.140	0.052	0.009	0.136	0.049	0.016	0.117	0.056	0.038	0.068	0.051	0.203
R^2^	0.201			0.120			0.124			0.196		
R^2^ change in step 1	0.064		0.000	0.020		0.027	0.054		0.000	0.084		0.000
R^2^ change in step 2	0.144		0.000	0.114		0.000	0.075		0.000	0.123		0.000
R^2^ change in step 3	0.022		0.072	0.018		0.180	0.026		0.053	0.018		0.157

B are standardized values; SE, standardized error; *p*, *p*-value.

## Data Availability

The datasets generated during and/or analysed during the current study are not publicly available due to privacy or ethical restrictions but are available from the corresponding author on reasonable request.
